# Efficacy of belimumab for severe childhood-onset systemic lupus erythematosus with diffuse proliferative glomerulonephritis: A case report

**DOI:** 10.1097/MD.0000000000034800

**Published:** 2023-08-25

**Authors:** Ying Hu, Jing Yuan, Bo Wang, Liang Ma, Yan Zha

**Affiliations:** a Department of Nephrology, People’s Hospital of Guizhou Province, Guiyang, China; b Division of Nephrology, West China Hospital of Sichuan University, Chengdu, China.

**Keywords:** acute pancreatitis, belimumab, childhood-onset systemic lupus erythematosus, lupus encephalopathy, lupus nephritis

## Abstract

**Introduction:**

Therapy of childhood-onset systemic lupus erythematosus (cSLE) with drugs is unsatisfactory. Some new drugs such as belimumab and rituximab may improve the course of severe cSLE, although there are few reports on treatment efficiency for these new drugs, especially belimumab.

**Case presentation:**

Here we report on a 16-year-old girl who was diagnosed with cSLE at the age of 13. After several immunosuppressive treatments, which included high-dose steroids, hydroxychloroquine sulfate, cyclophosphamide, etc for blood system damage, she showed little clinical improvement and developed severe pericarditis. Induction treatment with a combination of intravenous high-dose steroids, methylprednisolone, and cyclophosphamide was started, but, after 55 days, the patient developed lupus encephalopathy, lung infection, and lupus nephritis. After using high-dose steroids, cyclophosphamide, plasma exchange, gamma globulin, and appropriate anti-pulmonary inflammation drugs, treatment with tacrolimus was attempted but poorly tolerated by the patient and withdrawn. Eventually, in December 2019, belimumab was initiated on an off-label basis as a last resource to treat lupus nephritis. Belimumab was well tolerated by the patient and resulted in a rapid and marked improvement in clinical symptoms and reduction in proteinuria, serum complement levels and anti-double strand DNA antibodies titer; of note, the patient developed no infectious complications.

**Conclusion:**

Treatment with belimumab could result in prompt remission of severe cSLE with multiple organ damage without the pulmonary infection side effects for children deemed intolerant to conventional and second-line induction therapies. Belimumab should be considered as a potentially efficacious treatment in patients in severe childhood-onset systemic lupus erythematosus.

## 1. Introduction

Systemic lupus erythematosus (SLE) is an autoimmune disease with multiple system involvement. Approximately 15% to 20% of the cases occur in children. Childhood-onset systemic lupus erythematosus (cSLE), onset of disease before 18 years of age, is a more severe disorder than in adults and is accompanied by lupus nephritis (LN), central nervous system involvement, and multiple organ damage. The manifestation of cSLE is complex with high morbidity and mortality and poor prognosis.^[1]^ Treatment of cSLE accompanied by most severe form of kidney disease, diffuse proliferative glomerulonephritis, is challenging. The drugs for standard treatment are based on glucocorticoids and immunosuppressive agents, such as high-dose steroids in combination with a second agent (cyclophosphamide or mycophenolate mofetil [MMF]), which aim at limiting the T-cell derangement, with consequent B-cell activation and autoantibody production.^[2–4]^ Standard treatment can be complicated by a series of side effects, the most severe being serious infections. After clinical improvement, induction treatment is usually followed by a maintenance phase, where usually low-dose steroids plus an antiproliferative drug (MMF or azathioprine) are used.^[5]^ MMF is a powerful inhibitor of purine synthesis. Belimumab is a fully humanized monoclonal antibody that targets soluble B-lymphocyte stimulators (BLyS) and prevents BLyS from engaging its receptors on B cells. Belimumab is approved for treatment of serologically active SLE, and has been used in maintenance treatment after rescue induction treatment.^[6]^ However, the treatment efficiency for severe cSLE has been unknown.

## 2. Case report

We present the case of a 16-year-old girl who was diagnosed with SLE at the age of 13. She initially presented with multiple swollen lymph nodes, low-grade fever, facial rash, hypertension, and photophobia symptoms; serum antinuclear antibodies (ANA) and anti-double strand DNA antibodies (anti-dsDNA) were positive at high titer, and there was a marked reduction in serum complement fractions, and normal serum creatinine. Kidney biopsy was not performed, and the patient was treated with steroids 1 mg/kg/day, oral hydroxychloroquine, MMF, oral amlodipine besylate, and irbesartan tablets. The symptoms gradually improved.

**Figure 1.**
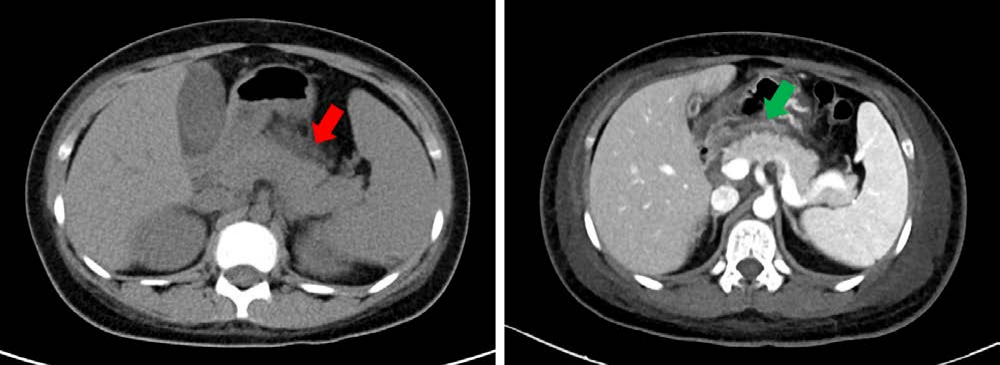
CT scan of the abdomen showed acute pancreatitis and hydrops abdominis, as shown by the red and green arrows.

Of note, she had irregular follow-up visits for 2 years after she was discharged from the pediatric department. In December 2018, she was hospitalized at the kidney clinic of our hospital for severe abdominal pain. Physical examination showed severe pitting edema in both lower limbs. Serum ANA and anti-dsDNA were positive at high titer, and serum complement fractions were low. A CT scan of abdomen showed the appearance of acute pancreatitis (AP) (Fig. 1).

The SLE Activity Index was 16, a value consistent with severe flare of SLE. She was treated with 3 high-dose steroid pulses (300 mg/day) plus 1 cyclophosphamide pulse (200 mg), antibiotics, proton pump inhibitor infusion therapy, subsequent oral steroid tapering, oral hydroxychloroquine, and MMF. The symptoms gradually improved.

**Figure 2.**
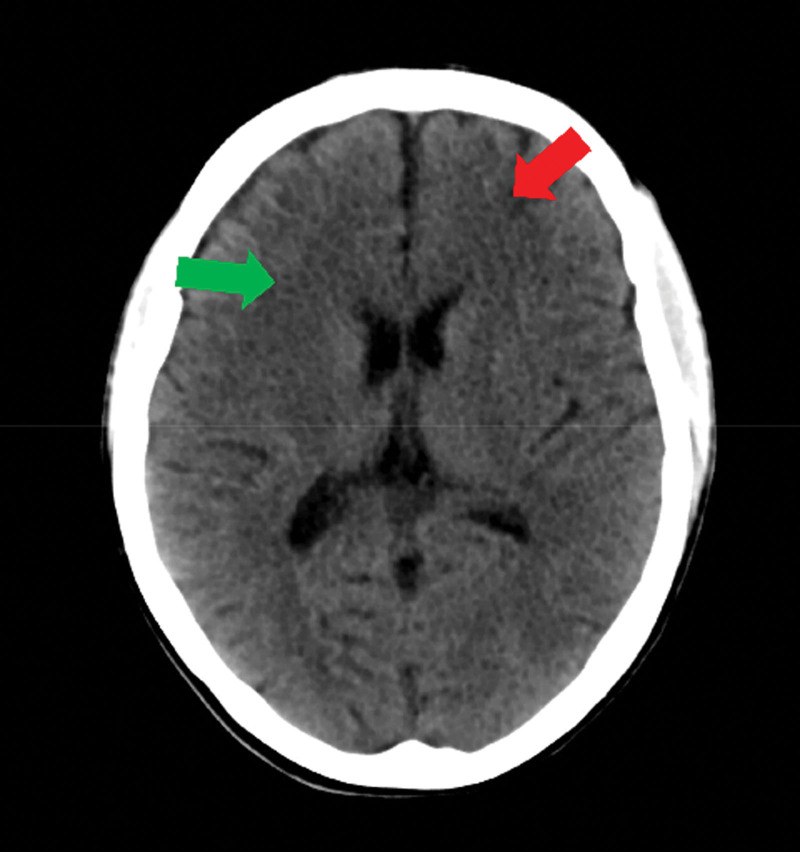
CT scan of the head showed multiple low-density foci in the bilateral frontotemporal as shown by the red and green arrows.

At 2 weeks after discharge, however, she was hospitalized for edema. Examination of blood and urine showed that blood albumin was 18.6 g/L and 2 + proteinuria on dipstick. Echocardiography showed heavy pericardial effusion. Kidney biopsy again was not performed. She was treated with 3 high-dose steroid pulses (methylprednisolone 300 mg) again; steroids were maintained at a dosage of 1 mg/kg/day of prednisone), and subsequent treatment included 1 cyclophosphamide pulse (0.4 g/2 weeks), oral hydroxychloroquine (0.1 g b.i.d.) and MMF (0.5 g b.i.d.). Two weeks later, a CT scan of the chest showed that she had a pulmonary bacterial infection, which prompted withdrawal of MMF, and calcineurin inhibitor (tacrolimus) was administered (2 mg b.i.d.). However, after 3 months, she was hospitalized for convulsions and disturbance of consciousness. Physical examination showed a great degree of muscle tension in the limbs. Cerebrospinal fluid examination findings were normal. Intracranial pressure was negative. A CT scan of the head showed multiple low-density foci in her bilateral frontotemporal parietal lobes (Fig. 2). Blood routine showed that hemoglobin (Hb) was 64 g/L. The SLE Activity Index was 18.

**Figure 3.**
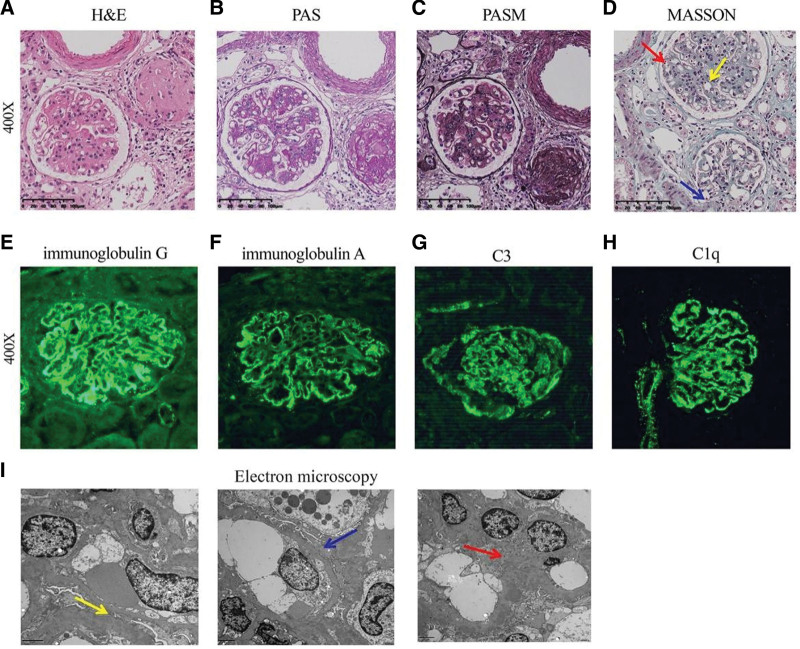
(A) Light microscopy: (A) hematoxylin and eosin (H&E) staining showed two globally sclerotic glomeruli, (B) the use of periodic acid-silver methenamine (PASM) stain showed one small-cell fibrous crescent formation, (C) periodic acid-schiff staining showed moderate proliferation of glomerular mesangial cells and stroma, and (D) Masson trichrome staining showed mesangial area, subepithelial, subendothelial eosinophil deposition. (All these pictures magnified 10 times). (B) Immunofluorescence: (E) immunofluorescence for immunoglobulin G, (F) immunoglobulin A, (G) complement C3, and (H) complement C1q revealed fused granular and lumpy deposits along the capillary loop and mesangial area. (C). Electron microscopy: Electron microscopy findings showed irregular thickening of the glomerular basement membrane. Electron-dense deposits along the subepithelial, inner basement membrane and subendothelial and mesangial area.

After multidisciplinary discussion, she was diagnosed with lupus encephalopathy and treated with 3 gamma globulin (20 g/day) and 3 high-dose steroid pulses (methylprednisolone 1000 mg), 2 plasmapheresis treatments, oral hydroxychloroquine and MMF, oral sodium valproate (0.5 g b.i.d.). Of note, high grade proteinuria persisted. In December 2019, the patient had a kidney biopsy. Histological analysis by light microscopy revealed 2 globally sclerotic glomeruli, 1 small-cell fibrous crescent formation, moderate proliferation of glomerular mesangial cells and stroma, and mesangial area, subepithelial, subendothelial eosinophil deposition (Fig. 3A); Immunofluorescence for immunoglobulin G, immunoglobulin A, complement C3, and complement C1q revealed fused granular and clumpy deposits along the capillary loop and mesangial area (Fig. 3B); Electron microscopy results showed the irregular thickening of the glomerular basement membrane and electron-dense deposits along the subepithelial, inner basement membrane and subendothelial and mesangial area (Fig. 3C).

The findings were consistent with diffuse hyperplasia and sclerosing LN with membranous LN (class IV-(A/C) + V ISN-RPS classification).^[7]^ Given the very poor renal function and overall prognosis in LN if left untreated, the use of belimumab as a last resort was deemed reasonable since belimumab was recently approved by the US Food and Drug Administration for children with SLE between 5 and 17 years of age.^[8]^ The patient was started on biweekly belimumab infusion at a dose of 10 mg/kg while being maintained on prednisone 8 mg/day. After 1 month of treatment, belimumab was switched to monthly administration and steroid tapering was begun. Belimumab was well tolerated by the patient and the symptoms gradually improved; of note, the patient remained free from infections. Kidney function remained normal, and proteinuria and hematuria gradually decreased. The anti-dsDNA antibody titer diminished rapidly and steadily; serum complement levels (which were already lower before belimumab initiation) normalized. After 10 months of treatment with belimumab, the patient was asymptomatic, with a serum creatinine of 44 µmol/L, 2 + hematuria and proteinuria on dipstick, C3 63 mg/dL, and C4 16 mg/dL, and anti-dsDNA was negative. Hemoglobin levels and blood albumin normalized almost completely. The systemic lupus erythematosus disease activity index (SLEDAI) score was 8, consistent with mild SLE activity. Because of the fair improvement, steroids currently are being tapered.

## 3. Discussion

Childhood SLE is often more severe than that in adults, especially when accompanied by LN. In the absence of appropriate treatment, the child may die from the disease or progress rapidly to renal failure.^[9]^ Moreover, the treatment of severe cSLE with active LN is challenging in children who do not respond to first-line treatments or those who develop severe infectious complications. Before the introduction of immunosuppressive therapy, the prognosis for LN was extremely dismal.^[10]^ In our case, the patient was diagnosed with cSLE at 13 years old, and the initial induction therapy was effective. Hydroxychloroquine as a DMARD was used in all the treatment process in order to prevent the recurrence of the disease. However, a kidney biopsy was not performed because she did not have regular follow-up. This might have accelerated the development of SLE. The patient suffered from AP, lung infection, lupus encephalopathy, and diffuse proliferative and membranous LN after she was diagnosed with cSLE. The cSLE Activity Index was 18. Undoubtedly, the patient had the severe type of SLE.

Unlike previous reported cases, the patient described here developed severe cSLE, including AP, lupus encephalopathy, diffuse proliferative and membranous lupus nephritis, which was most likely attributable to the active cSLE. Although AP might be a side effect of high-dose steroids, no further abdominal pain episodes occurred after the high-dose steroid treatment. This suggested that severe AP was also a clinical manifestation of active cSLE. Some studies reported that the main etiologies for AP in lupus patients were disease activity, corticosteroid use, infectious diseases and hypertriglyceridemia. However, the most important cause of pancreatitis in lupus patients was disease activity (76%–84%).^[11–13]^ The treatment for AP in lupus patients is mainly based on intravenous methylprednisolone, as evidenced in our case. Although the treatment was advanced, the patient still had lupus encephalopathy. The SLEDAI was 18. Levels of C3 and C4 continued to be low. Histological analysis revealed diffuse hyperplasia and sclerosing LN with membranous LN (class IV-(A/C) + V ISN-RPS classification).

Fortunately, timely initiation of belimumab treatment protected the patient from progressing to multiple organ failure after the induction therapy, which included 3 gamma globulin (20 g/day) and 3 high-dose steroid pulses (methylprednisolone 1000 mg), 2 plasmapheresis treatments, oral hydroxychloroquine, and MMF. Astoundingly, the patient made a prompt recovery following belimumab treatment without need for frequent hospitalization or intensive supportive care. The successful experience in this case suggests that timely treatment with belimumab is worth trying after t induction therapy, especially in those critically ill patients with multiple organ damage, because it may significantly improve the prognosis.

This case report sheds new light on a therapeutic strategy for this potentially fatal disease. Belimumab is a monoclonal IgG antibody targeting soluble BLyS and aimed at lowering available BLyS levels for autoreactive B-cell selection and survival. Belimumab was approved in recent years for SLE treatment and notably it is the only drug approved for SLE in the last 60 years, since the time corticosteroids, hydroxychloroquine, and nonsteroidal anti-inflammatory drugs were approved for SLE.^[14]^ Two international phase III trials, BLISS-52 and BLISS-76 (ClinicalTrials.gov identifiers NCT00424476 and NCT00410384, respectively) evaluated the safety and efficacy of belimumab in patients with autoantibody-positive (seropositive) SLE (defined as a serum ANA titer of 1:80 and/or positive results on a test for serum anti-dsDNA). Both trials showed that belimumab 10 mg/kg plus standard therapy for SLE (including corticosteroids, immunosuppressive agents, and/or antimalarial agents administered alone or in combination) was generally well tolerated and met the primary endpoint of significant disease improvement.^[15–18]^ In our case, belimumab is thus far well tolerated by our patients, with the absence of notable side effects or adverse events. Belimumab resulted in a strong improvement in parameters related to disease activity such as C3 and C4 and a consistent reduction in proteinuria, microhematuria, and SLEDAI score. We hope that future ongoing clinical trials^[17]^ may establish a clear role for belimumab in the treatment of LN and better identify those patients who may benefit the most from this treatment. Moreover, we suggest that belimumab may be used off-label in that subset of patients with active LN who develop severe lupus encephalopathy after first- and second-line conventional treatments for LN or those who are intolerant to them.

Several questions remained unanswered, however. First, the contribution of treatment with cyclophosphamide pulse (cyclophosphamide 0.4g biweekly) and high-dose steroid pulses (methylprednisolone 1000 mg) cannot be completely ignored as having a role in the patient’s quick remission in a short time when the patient was had severe lupus encephalopathy, because steroids and cyclophosphamide were used throughout the course of treatment. They are the classic treatment for severe SLE. Moreover, 3 gamma globulin pulses (20 g/day) improved the patient’s resistance at this critical moment. Second, the contribution of 2 plasmapheresis treatments cannot be excluded because this treatment eliminated a large number of inflammatory factors in the patient’s body.

## 4. Conclusion

A 16-year-old girl diagnosed with cSLE at age 13 presented with AP, serious lupus encephalopathy, and severe LN and was deemed intolerant to conventional and second-line induction therapies. Belimumab treatment resulted in a marked improvement without pulmonary infection side effects. Successful remission in this case indicates that belimumab should be considered as a potentially efficacious treatment in patients with multiple organ damage in cSLE who cannot tolerate conventional therapies.

## Acknowledgments

We acknowledge Jing Yuan for her valuable help in the follow-up of the patient.

## Author contributions

**Data curation:** Ying Hu.

**Formal analysis:** Jing Yuan, Yan Zha.

**Investigation:** Yan Zha.

**Methodology:** Jing Yuan.

**Project administration:** Liang Ma.

**Resources:** Ying Hu.

**Software:** Bo Wang.

**Supervision:** Yan Zha.

**Writing – original draft:** Ying Hu.

**Writing – review & editing:** Bo Wang, Liang Ma.
